# Experiences of spinal cord injury patients admitted to the rehabilitation unit at the national referral hospital in Khomas region, Namibia

**DOI:** 10.4102/ajod.v11i0.1018

**Published:** 2022-07-27

**Authors:** Daniel O. Ashipala, Lettie Langendorf

**Affiliations:** 1 Department of General Nursing Science, School of Nursing and Public Health, Faculty of Health Sciences and Veterinary Medicine, University of Namibia, Rundu, Namibia

**Keywords:** spinal cord injury, rehabilitation, challenges, lived experiences, patients, re-integration

## Abstract

**Background:**

Spinal cord injury (SCI) remains one of the major causes of disability globally. It results in permanent physical disability secondary to devastating neurological defects. When a person sustains SCI, substantial changes and challenges in their lives occur, regardless of their age or socioeconomic status. In Namibia, the knowledge on SCI experiences could be used to improve the care rendered to patients with this injury.

**Objectives:**

The purpose of this study was to explore and describe the experiences of patients with SCIs admitted to the rehabilitation unit at the national referral hospital in Khomas region, Namibia.

**Method:**

A qualitative, explorative and descriptive study design was used. Data were collected by means of in-depth, semi-structured, face-to-face interviews with 15 participants from the rehabilitation unit. Data were analyzed using thematic analysis.

**Results:**

Analysis of the data in this study identified three themes: negative experiences, positive experiences and measures to improve the lives of people living with SCI in the community. Participants experienced varied emotions from anger, stress, disbelief, frustration and sadness, which led to depression. In addition, participants experienced discrimination due to lack of community acceptance hence, improving awareness remains key.

**Conclusion:**

This study provided insight into the lived experiences of those living with SCI as they narrated their struggle from the onset of SCI to their reintegration into the community. The study’s findings can be used to develop self-care strategies and ongoing interventions that focus on maintaining physical and psychological health for spinal-cord injured persons throughout the course of living with disability.

## Introduction

Spinal cord injury (SCI) is a devastating, life-threatening condition that affects every aspect of life, including the physical, social and psychological health of the affected people. The World Health Organization (WHO) (World Health Organization 2013) reported that around 500 000 people sustain an SCI each year, and these people are two to five times more likely to die prematurely, with the worst survival rates in low-income and middle-income countries. In addition, the WHO reported that men are most at risk of SCI between the ages of 20 and 29 years and 70 years and older, whilst women are most at risk between the ages of 15 and 19 years and 60 years and older. Furthermore, the WHO also reported that 90% of causes of SCI are because of trauma such as road traffic accidents, falls and violence. Road traffic injuries place a burden on global and national economies and household finances. The Namibia Statistics Agency (2016) further reported that many families are driven into poverty by the loss of income and the added burden of caring for the family members who become disabled because of such injuries. The *New Era* (2014) reported that according to the WHO, Namibia is ranked first in the world in terms of the number of road deaths per 100 000 residents. Although Africa accounts for just 2% of registered vehicles, the continent is responsible for about 16% of annual global road deaths. The *New Era* (2014) further reported that a recent study on mortality from road crashes in 193 countries by Schoettle and Sivak (2014), from the University of Michigan Transportation Research Institute, indicated that in Namibia, about 53% of the population are more likely to die in a vehicle collision than from cancer or other diseases.

According to the Namibia Statistics Agency (2016), in 2018 the road network comprised 45 387 km, of which approximately 14% was tarred. The 2016/2017 report of the Motor Vehicle Accident (MVA) Fund of Namibia indicates that 3044 people were injured in 1772 car crashes on national roads across the country. The report further reveals 528 road crashes and 965 injuries for the festive season during that period, indicating a reduction from the 645 crashes (18%), 1292 injuries (25%) and fatalities (5%) recorded during the 2016/2017 festive season from the previous year.

Records at the national referral hospital rehabilitation unit at the Windhoek Central Hospital in Khomas region indicate that the most common causes of SCI are MVAs, which account for almost 18%. About 13% result from acts of violence, commonly knife and gunshot wounds (Hedimbi, Amakali-Nauiseb & Niikondo 2019). Statistically, Chatukuta (2020) observed that 60% of SCIs result from recreational activities, such as diving into shallow water, as well as cultural and traditional sports activities; 1% results from falls, mostly by senior citizens from the age of 65 and above. The researcher consequently considered it imperative to study the lived experiences of people with SCIs at the national referral hospital, as this is the primary care facility for those with traumatic spinal cord injury (TSCI) in Namibia.

Although many studies have been conducted on SCI, most of these focused on the availability of wheelchairs, accessibility to primary healthcare and the psychological effects of patients with SCI (Paulus-Mokgachane, Visagie & Mji 2019; Pefile, Mothabeng & Naidoo 2019; Sefotho, Jabulani Mpofu & Maree 2017; Visagie, Duffield & Unger 2015; Visagie et al. 2015). In Namibia, the Disability Resource Centre offers vocational trainings to people with disabilities and provides peer and entrepreneurial support to those who want to start their own business (Shumba & Moodley 2018). In Namibia, the National Disability Council is the custodian in enforcing the Disability Act, 2004. The National Disability Council advocate for the rights of people with disability in Namibia as provided for in the Disability Act, 2007 according to Chichaya, Joubert and McColl (2018). As such, additional research in the Namibian context is necessary to explore the experience of patients with SCI. Whilst literature on the experience of with SCI has increased globally, this has not been the case in Namibia.

Studies exploring the in-depth experiences of patients with SCI are necessary in order to devise coping strategies that can mitigate these challenges. This study is amongst few that examine the experiences of patients with SCI. In addition, this research sought to understand the experiences of patients with SCI. Researching these experiences could contribute to the development of self-care strategies and ongoing interventions that focus on maintaining physical and psychological health for both spinal cord injured persons and their family members throughout the course of living with the disability.

### Research question

What are the experiences of patients with SCI admitted to the rehabilitation unit at the national referral hospital in Khomas region, Namibia?

### Research aim

The aim of this study was to explore and describe the experiences with SCI admitted to the rehabilitation unit at the national referral hospital in Khomas region, Namibia.

## Research methodology and design

The study used a qualitative, exploratory and descriptive design. According to Hennink, Hutter and Bailey (2020), qualitative studies focus on the meaning and understanding of experiences as lived by participants. An explorative research design involves the identification of problems within a certain practice and justification of that practice (Flick 2018). The inquiry process involved the researchers conducting the study in a natural setting and developing a complex holistic picture, as well as analysing and reporting the detailed views of the participants (Cardano 2020). This study focuses on the meaning and understanding of experiences as the participants experienced them. The goal of qualitative research is to understand rather than explain or predict (Mohajan 2018). Semistructured individual interviews were conducted to enable the researchers to understand the participants’ experiences.

### Setting

The study was conducted at a national referral hospital in Khomas region, Namibia. The SCI rehabilitation unit, Spinalis, which opened in 2013, is the country’s first SCI unit. After patients are discharged from the general ward, they are referred to the rehabilitation unit, which receives referrals from all 14 regions. The rehabilitation programme ranges between 8 and 12 weeks depending on the type of injury. Between 2016 and 2018, the eight-bed rehabilitation unit recorded an admission of 147 with disability resulting from TSCI. The training provided during rehabilitation comprises basic but complex aspects that facilitate physical therapy, skill-building activities and counselling to provide social and emotional support, as well as to increase the quality of life for everyone. The programme promotes maximising the level of independence before returning patients to their previous social and working conditions. The unit consists of a comprehensive rehabilitation team of multidisciplinary health professionals, consisting of doctors and nurses, an occupational therapist, a physiotherapist, a social worker and a rehabilitation coach.

### Population and sample

The population of this study comprised 20 patients with TSCI at the national referral hospital. The researcher conveniently selected 15 patients from the population of 20 patients who have completed their rehabilitation programme, who came for follow-up during August and September 2020. Inclusion criteria included patients with SCI who were willing to participate by signing an informed consent, with the ability to communicate verbally, who were over the age of 18 years and had completed the rehabilitation programme. The researcher kept a reflexive journal of all the information obtained from the beginning of the study, including personal reflections in relation to the study. The only data that the researcher used was from audio recordings, transcripts and field notes in the analysis and did not add any other information not provided by the participants. The independent coder was given the researcher’s reflexive diary, which was kept throughout the research process and contained all field notes, including intuitive notes that assisted in bracketing.

### Data collection

In this study, data were collected during August and September 2020 after approval had been granted by the School of Nursing Ethics Review Committee and the Ministry of Health and Social Services Institutional Review Board. The semistructured interviews were conducted by the same interviewer for all patients in accordance with an interview guide, which was developed based on the research question and the literature review. The interviews were conducted in order to capture the participants’ experiences pertaining to living with SCI. The interview guide comprised the following sections: section A and section B. Section A requested sociodemographic information from the participants to contextualise the research findings in line with their background information. Section B consisted of two questions. The first question was about the lived experience after sustaining an SCI, followed by probing questions. The second question was about what could be done to improve the life of a person living with an SCI. Data saturation was reached at 15 participants and no further interviews were conducted. Each interview lasted between 30 min and 34 min.

The following interview questions were posed:

- What are your experiences as a patient with an SCI admitted to the rehabilitation unit at the national referral hospital in Khomas region, Namibia?
- What can be done to improve the lives of patients living with SCI who are admitted to the rehabilitation unit at the national referral hospital in Khomas region, Namibia?

### Data analysis

Data from the audio recordings were transcribed verbatim before being analysed using qualitative thematic analysis (Braun et al. 2018). The transcripts and narratives were thematically analysed following Braun’s six-step method of data analysis, which included: (1) organising and preparation of data, (2) developing a sense of all the data, (3) coding data following Tesch’s nine steps, (4) identifying and describing themes, (5) representing the findings and (6) interpreting the data (Braun et al. 2018). The researchers and the independent coder then held a consensus discussion and agreed on the main themes and subthemes that emerged. The analysed data were presented using the main research questions and objectives as the key themes.

### Measures to ensure trustworthiness

Trustworthiness in this study was ensured through the application of the credibility, transferability, dependability and conformability criteria proposed by Kyngäs, Kääriäinen and Elo (2020). Credibility in this study was assured during the two months that the researcher spent with the participants until data saturation was reached, thus gaining an in-depth understanding of the phenomenon. Prior to the actual data collection, testing of the interview guide was conducted on three participants who were also part of the study; however, no changes were made to the interview guide. The researcher has ensured transferability through a thick description of the data collection process, using verbatim transcriptions of the voice recordings and field notes. Dependability was facilitated by means of literature control and prolonged engagement (Nguyen et al. 2021). Confirmability was assured by recording the interview to ensure as far as possible that the study findings were the results of the respondents’ experiences and ideas. This study used an independent coder to confirm the data collected via the interview guide, which in turn were confirmed by the researchers to make sure that the data were accurate and in line with the information provided by the participants.

### Ethical considerations

The research was conducted after approval was granted by the School of Nursing Research and Ethics Committee of the Faculty of Health Sciences, University of Namibia, Namibia (reference no. SoNREC 47/2020), whilst permission to conduct the study and access participants was obtained from the Research Committee of the Ministry of Health and Social Services (reference number LL2020). Participants’ right to privacy, the right to anonymity and confidentiality, the right to fair treatment and the right to protection from discomfort and harm were considered throughout the process (Hays & McKibben 2021). Informed consent was sought verbally from the participating respondents and through their signing of a consent form, as well as permission to collect field notes and to use an audio recorder, was obtained from the participants. Participation in the study was entirely voluntary and participants had the right to withdraw at any given time without any penalties. Interviews were conducted in an office in the unit to ensure privacy. Data were kept in a computer encrypted with a password known only to the researchers. Furthermore, only those questions relevant to the aims and objectives of the study were asked. Instead of names, numbers were used during the interviews to prevent the linking of data with participants’ identities.

### Participants’ demographical data

The participants in this study comprised 15 adults (10 males and 5 females) aged between 20 and 59 years, all of whom had complete SCI resulting in disability (i.e. four quadriplegics and 11 paraplegics). Six of the participants were married, five were cohabiting and four were single. Nine participants had children. All participants had formal education and the majority had tertiary education. The main source of income for nine of the participants was from a social grant, whilst six were employed prior to the injury. The sociodemographic data of the participants are given in Table 1.

**TABLE 1:** Characteristics of participants.

Variable	Frequency
**Age**	
20–29	5
30–39	4
40–49	4
50–59	2
**Gender**	
Male	10
Female	5
**Marital status**	
Single	4
Married	6
Cohabitating	5
**Education**	
Primary	4
Secondary	5
Tertiary	6
**Source of income**	
Employed	6
Social grant	9
**Have children**	
Yes	9
No	6
**Level of injury**	
Cervical	4
Thoracic	11

## Presentation and discussion of findings

The themes that emerged from the data analysis are indicated in Table 2 as follows: (1) negative experiences, (2) positive experiences and (3) measures to improve the lives of people living with SCI in the community.

**TABLE 2:** Summary of findings.

Themes	Subthemes
1. Negative experiences	Limited accessibility Emotional stress leading to depression Societal discrimination against wheelchair users That look of pity from society
2. Positive experiences	Availability of technical aids and assistive devices Gaining knowledge in self-grooming and self-care activities Family equipped with knowledge of care for rehabilitation
3. Measures to improve the lives of people living with SCI in the community	Outreach programmes for SCI awareness Peer support group for support and motivation Rehabilitation services to be decentralised to the regions Promotion of the rights of people with disabilities

SCI, spinal cord injury.

### Theme 1: Negative experiences

This theme is a description of the participants’ experiences regarding living with SCI. The following subthemes were identified for this theme: (1) limited accessibility, (2) emotional stress leading to depression, (3) societal discrimination against wheelchair users and (4) society feeling pity.

#### Subtheme 1: Limited accessibility

Limited accessibility was an issue expressed by all participants. Following rehabilitation, some patients became independent, but the issue of accessibility became the new reality for most, starting with the limited access to buildings and modes of transport such as taxis and buses, which did not accommodate them. Although they could afford the fares, they still could not access this type of transport. Problems of access were expressed as follows by a number of the participants:

‘Having to go to a place that isn’t accessible really breaks me down, or even going to family functions where I don’t have access to enter, I end up feeling like I am a burden because now they have to spend time trying to accommodate you.’ (P7, 28 year old, Male)

‘People do not consider the problems of wheelchair users. You will find that the bathroom stalls are too small for my wheelchair to enter, and this is an issue also for many other places, such as lodges, restaurants, offices etc. They are really not wheelchair friendly.’ (P3, 20 year old, Female)

Mobility is perceived as one of the most restricted domains of social participation following an SCI. The lack of appropriate access to buildings and transport remains an almost unbearable experience. These results correspond with the work of Sale et al. (2016) who found that the dependency of people with an SCI on other people devalues their existence as human beings, leading to a lack of personal privacy, which really defines the personhood of human beings.

#### Subtheme 2: Emotional stress leading to depression

The participants expressed disbelief at what had happened to them, stating that their grief was not only about the physical loss caused by the SCI, but that it also had an emotional aspect that contributed to the depression they experienced.

Grieving for the loss of personal control and independence after SCI led to depression, a negative psychological state in which participants went through a deep sadness, apathy and lethargy of emotions in relation to the SCI, resulting in profound feelings of anger, injustice and unfairness. Some of the participants had this to say:

‘After I was told by the doctor that I would never walk again, all sorts of things went through my mind; I even thought of killing myself, because I thought my life would be meaningless.’ (P10, 40 year old, Male)

‘I was really sad and went into depression; I cried a lot, and I kept my feelings to myself because I thought no one would understand what I am going through. I hated my wheelchair and I thought dying would be a better option.’ (P1, 22 year old, Female)

The findings of this study support those of Hatefi et al. (2019), who found that emotional stress for people who have experienced injuries is common. These findings are also supported by Torabian, Sabet and Meschi (2019), who observed that patients who have sustained an SCI often become emotionally weak and have traumatic depression, leading to suicidal thoughts if not managed properly in the rehabilitation process.

McDonald, Pugh Jr and Mickens (2020) again confirmed that people with SCIs are at increased risk of developing major depressive disorder, as well as other negative psychological effects during the rehabilitation phase or after returning to live in the community.

#### Subtheme 3: Society discriminates against wheelchair users

Societal discrimination against people with SCI has a detrimental effect on the achievement of holistic rehabilitation (Kisala et al. 2015). Some of the participants mentioned that although they were dealing with this unfortunate situation, one of the factors that made their life more miserable was the reaction from society, which made it even more difficult to reintegrate into the community. This is evidenced by the following responses:

‘I got very emotional, especially after I lost my friends; there were those who believed I could not socialise with them the way I used to; they would call me boring, and this really got to me.’ (P15, 47 year old, Male)

‘Society treats you as if you are unable to do anything; they do not respect you and see you as nothing, don’t allow to participate in decision-making and they would not want you to represent the community.’ (P15, 47 year old, Male)

These findings are echoed by Russell et al. (2021), who found that lack of physical ability to perform routine duties generally has a detrimental effect on the socialisation process of those who have sustained an SCI. Other studies, such as McDonald et al. (2018), had shown that when victims of SCI realise that they can no longer perform as they used to, as a result of the reception they receive from society, they may remove themselves from society.

#### Subtheme 4: Society feeling pity

Participants indicated how much they had been the object of society’s pity and indicated that this pity disrupted their reintegration into the community:

‘When my friends came to visit me and start to cry for my sorry state, some kept their distance because they were not used to seeing someone suffer from SCI’. (P9, 35 year old, Male)

‘The look of pity in people’s eyes made me not want to be outside, because I did not like the feeling of pity towards me.’ (P6, 40 year old, Male)

These findings are supported by Burkhart, Skemp and Siddiqui (2021), who stated that social constructs of disability, even if in the form of positive philanthropy, affect the disabled person as they view themselves as being more than just disabled.

### Theme 2: Positive experience

This theme focuses on the positive experiences of those living with an SCI. Based on the data collected, the following subthemes were generated: availability of technical aid and assistive devices; gaining knowledge on self-grooming and self-care activities; family equipped with knowledge of care for rehabilitation.

#### Subtheme 1: Availability of technical aid and assistive devices

The availability of specialised SCI management and the provision of technical aids are considered as necessary to help patients with SCI adapting to their new lifestyle. Accordingly, some of the participants had this to say:

‘There are more advanced techniques to do things differently than before, so I think that it also helps me a lot.’ (P11, 45 year old, Male)

‘I go where I want to go with the help of my wheelchair, because they are now my legs, and I am grateful that I can drive myself.’ (P4, 49 year old, Male)

These findings are supported by Yoo et al. (2019), who maintained that assistive devices are important not only in moving patients with SCI around, but also for routine self-care and personal tasks such as eating and bowel and bladder management. These findings are supported by Manns, Hurd and Yang (2019), who found that with modern technology, it is becoming easier for people with disabilities to improve their performance of certain activities, routines and socialisation.

#### Subtheme 2: Gaining knowledge of self-grooming and self-care activities

Learning appropriate techniques for self-care is important and an explicit goal of SCI rehabilitation. Accordingly, a common response from all the participants was that after undergoing rehabilitation, they had gained functional independence at the level of their ability:

‘I saw that there are things one can do without being dependent. I can now drive also, which [*is*] something I couldn’t do in the beginning, and I never thought it would be something possible.’ (P4, 49 year old, Male)

‘I am trained to become independent to the level of my ability, for example, how to bath[*e*], how to transfer self from a wheelchair to any other place.’ (P7, 28 year old, Female)

A study by Mir et al. (2019) confirmed that rehabilitation is the strongest weapon to counter the internal and external challenges facing people with disabilities. The inability to perform an everyday task can lead to a loss of self-worth; hence, rehabilitation is often described as promoting inner strength. It has been observed in the findings of this study that despite the adverse consequences of SCI, patients still experienced some positives in adjusting to their new lives. This finding is supported by Qasheesh et al. (2021), who found that rehabilitation after SCIs helps to improve the adaptability and adjustment of patients with such injuries.

#### Subtheme 3: Family equipped with knowledge of care for rehabilitation

Support on the part of family and friends has an influence on the building of a strong self. Their involvement has a positive effect on the recovery of a person with an SCI, as is indicated by the participants’ responses:

‘Because my wife received caregiver training, she has always been very supportive, and my family is always around to assist.’ (P12, 50 year old, Male)

‘My injury has left me not be able to control my bladder and bowel; my wife and my two daughters are sharing the responsibilities to care for me.’ (P5, 58 year old, Male)

A study by Conti et al. (2020) confirmed that families are the determinant factors that shape a continuous rehabilitation process. Similarly, McDaid et al. (2019) observed that families are the primary aspect of therapy, therefore equipping them with information is necessary to help people with SCI to adjust to a new life.

### Theme 3: Measures to improve the lives of people living with spinal cord injury in the community

This theme is a description of what participants mentioned when they were asked to suggest actions to be completed in the future that are required to improve the lives of people with SCI in the community. The subthemes in this theme are as follows: outreach programmes for SCI awareness, peer support groups for support and motivation, rehabilitation services to be decentralised to the regions and promotion of the rights of people with disabilities.

#### Subtheme 1: Outreach programmes for spinal cord injury awareness

Participants expressed the need for outreach programmes to create awareness of SCI in the community. Such programmes provide specialist knowledge, advice, early intervention and education for patients with SCIs, their families and the community. Participants had the following to say on this topic:

‘To create awareness in the community, for those who have no knowledge about people living with disabilities, and to encourage people with disabilities to be more active and socialise more.’ (P5, 58 year old, Male)

‘More outreach programmes to create awareness on SCI and break the chain of stigmas about those with spinal cord injuries.’ (P3, 20 year old, Female)

Mobile teams could play an important role in providing adequate follow-up and maintaining long-term health (Divanoglou et al. 2018). Merritt et al. (2019) described an attempt to reach people in rural villages in India, which they called Paraplegia Safari, as having a positive effect.

#### Subtheme 2: Peer support group for support and motivation

Participants reported that peer support groups are an important aspect, helping them through the recovery process. Peer mentors and support groups provide a picture of what is possible and in such groups helpful tips gained from experience are shared. The following responses were observed from the participants:

‘I know a few wheelchair users who had their accident way before me and had experienced way more than me, so when I need help or advice, I will go to them. Peer group is very important, and it has helped me so much.’ (P14, 32 year old, Male)

‘Forming a group like an association where people with spinal cord injuries can come together to motivate and support each other to reach better outcomes in their lives.’ (P7, 28 year old, Female)

These findings are supported by those of Ronca et al. (2020), who indicated that peer support groups help raise awareness of SCI programmes. If such programmes were intensified, those with peer support would see more improvement in their depression and patients with SCI would accept their disability, take better care of themselves and feel more satisfied, in control and optimistic.

#### Subtheme 3: Rehabilitation services to be decentralised to the regions

The participants highlighted the need for rehabilitation services in the regions. These specialised services are crucial for reducing the possible complications that may arise. Participants had this to say:

‘More wards such as Spinalis (7west) should be created countrywide for rehabilitation, because without rehabilitation, people with SCI become burdens.’ (P6, 40 year old, Male)

‘They do not have a centre for rehabilitation, a place where those with SCI can come together and socialise with each other, exercise with other people with SCI. So if the rehabilitation centre can be properly decentralised to towns, it would make life easier for people to learn about the issues those with SCI have.’ (P4, 49 years old, Male)

The findings have shown that participation in rehabilitation enhances the likelihood of improving quality of life after an SCI. These findings are supported by those of Merritt et al. (2019), which show that people with SCIs need special services to facilitate their participation in the community and to improve their quality of life.

#### Subtheme 4: Promotion of the rights of people with disabilities

The participants mentioned that it serves no purpose to have good policies on paper; if they are not implemented, they will have no benefit for society. The constraints experienced by the participants are a result of a lack of services and policies that facilitate a successful outcome and reintegration into society for people with SCIs. The participants had this to say in this regard:

‘Namibia has signed the UN convention, and there are a lot of rights for people with disabilities.’ (P10, 40 year old, Male)

‘I think if we can just start by addressing each right at a time and implementing them.’ (P8, 25, Female)

The promotion of the rights of people with disabilities would give equal rights to those with SCIs to live in the community, also giving them choices equal to those of others. The improved survival rate and increased life expectancy amongst people with SCIs mean that they will live with the disability for relatively longer periods, giving rise to new problems and responsibilities that can negatively impact their lives (Houlihan et al. 2016).

### Limitations and areas for further research

This study only explored the lived experiences of people with TSCI at a national referral hospital who had undergone rehabilitation. In addition, the mixed role of the researcher (as both a clinician and as someone with whom the participants were familiar) assisted in building trust and making them feel comfortable. However, this familiarity could be seen as a limitation if the participants in any way felt obliged to participate or give answers to please the researcher or to answer in an expected way. The researcher kept a reflexive journal of all the information obtained from the beginning of the study, including personal reflections in relation to the study. The only data that the researcher used was from audio tapes, transcripts and field notes in the analysis and did not add any other information not provided by the participants. Although the findings cannot be generalised, there are still areas that require further study; for example, further research is required for an inferential study to measure the impact of psychological challenges on the lifespan of people with SCIs or a study to measure the impact of rehabilitation centres in countering challenges faced by people with SCIs. In addition, the impact of an SCI on the life of the primary family caregiver should be explored.

### Recommendations

The recommendations are based on the literature and the findings of this study. Accordingly, the researcher recommends the following:

- Outreach programmes for SCI awareness are needed. There is a need to engage in more outreach programmes to create awareness of SCI and break the stigmas attached to disability. Outreach programmes will support patients discharged from the hospital following an SCI, thus assisting to ensure a smooth transition into the community.
- Peer support groups for support and motivation are needed to train the newly affected patients with SCIs. Peer support plays an important role in supplementing the information provided by the multidisciplinary rehabilitation team. Role-modelling particularly helps those who may find it challenging to discuss their conditions.
- Rehabilitation services should be decentralised to the regions, as patients with SCIs experience challenges related to a lack of specialised services in the various regions after being discharged from rehabilitation. The concept of rehabilitation is relatively new in Namibia, therefore having decentralised rehabilitation services would be in line with the *National Health Policy Framework (2010 – 2020).* This policy serves as a fundamental principle for providing public health services to the population with the intention of giving access to a range of effective public health interventions.
- Promotion of the rights of people with disabilities needs to be made a priority in order to increase knowledge on disability rights amongst the citizens of Namibia, because the Namibian constitution guarantees the right to full and equal participation for all its citizens, including men, women and children with disabilities.
- Further research is required for an inferential study to measure the impact of psychological challenges on the lifespan of people with SCIs or a study to measure the impact of rehabilitation centres in countering challenges facing people with SCIs. In addition, the impact of an SCI on the life of the primary family caregiver should be explored.

## Conclusion

The purpose of this study was to explore and describe the lived experiences of people with SCIs at a national referral hospital in Khomas region, Namibia. This study provided an insight into the lived experiences of people with SCIs at the national referral hospital. It is worth noting that the participants indicated that they had both negative and positive experiences. Participants experienced varied emotions ranging from anger, stress, disbelief, frustration and sadness, which eventually led to depression. The study also highlighted the discrimination resulting from the failure on the part of society to accept and see people with disability as merely people with different abilities; hence, a need to improve awareness of SCI is much needed. Positive experiences, as highlighted by the participants, include the family being equipped with knowledge of care for rehabilitation, as well as family involvement. In addition, key rehabilitation factors that were highlighted in this study included the commitment of the family members of those with SCI and their role in ensuring constant care, especially for those with more debilitating injuries. The availability of technical aids and assistive devices plays a major role in helping those with SCI reintegrate into society, and these are especially important in giving them a sense of independence, helping them to gain knowledge on self-grooming and self-care activities.
